# The nonvesicular sterol transporter Aster-C plays a minor role in whole body cholesterol balance

**DOI:** 10.3389/fphys.2024.1371096

**Published:** 2024-04-17

**Authors:** Rakhee Banerjee, Rachel C. Hohe, Shijie Cao, Bryan M. Jung, Anthony J. Horak, Iyappan Ramachandiran, William J. Massey, Venkateshwari Varadharajan, Natalie I. Zajczenko, Amy C. Burrows, Sumita Dutta, Maryam Goudarzi, Kala Mahen, Abigail Carter, Robert N. Helsley, Scott M. Gordon, Richard E. Morton, Christopher Strauch, Belinda Willard, Camelia Baleanu Gogonea, Valentin Gogonea, Matteo Pedrelli, Paolo Parini, J. Mark Brown

**Affiliations:** ^1^ Department of Cancer Biology, Lerner Research Institute of the Cleveland Clinic, Cleveland, OH, United States; ^2^ Center for Microbiome and Human Health, Lerner Research Institute, Cleveland Clinic, Cleveland, OH, United States; ^3^ Department of Cardiovascular and Metabolic Sciences, Lerner Research Institute, Cleveland, OH, United States; ^4^ Department of Inflammation and Immunity, Lerner Research Institute, Cleveland Clinic, Cleveland, OH, United States; ^5^ Department of Physiology and the Saha Cardiovascular Research Center, University of Kentucky College of Medicine, Lexington, KY, United States; ^6^ Department of Internal Medicine, Division of Endocrinology, Diabetes, and Metabolism, University of Kentucky College of Medicine, Lexington, KY, United States; ^7^ Proteomics and Metabolomics Core, Lerner Research Institute, Cleveland Clinic, Cleveland, OH, United States; ^8^ Department of Chemistry, Cleveland State University, Cleveland, OH, United States; ^9^ Department of Laboratory Medicine, Karolinska Institute, Huddinge, Sweden

**Keywords:** cholesterol, lipoprotein, metabolism, steroid hormone, oxysterol

## Abstract

**Introduction:**

The Aster-C protein (encoded by the *Gramd1c* gene) is an endoplasmic reticulum (ER) resident protein that has been reported to transport cholesterol from the plasma membrane to the ER. Although there is a clear role for the closely-related Aster-B protein in cholesterol transport and downstream esterification in the adrenal gland, the specific role for Aster-C in cholesterol homeostasis is not well understood. Here, we have examined whole body cholesterol balance in mice globally lacking Aster-C under low or high dietary cholesterol conditions.

**Method:**

Age-matched *Gramd1c* ^+/+^ and *Gramd1c* ^−/−^ mice were fed either low (0.02%, wt/wt) or high (0.2%, wt/wt) dietarycholesterol and levels of sterol-derived metabolites were assessed in the feces, liver, and plasma.

**Results:**

Compared to wild type controls (*Gramd1c* ^+/+^) mice, mice lacking*Gramd1c* (*Gramd1c* ^−/−^) have no significant alterations in fecal, liver, or plasma cholesterol. Given the potential role for Aster C in modulating cholesterol metabolism in diverse tissues, we quantified levels of cholesterol metabolites such as bile acids, oxysterols, and steroid hormones. Compared to *Gramd1c* ^+/+^ controls, *Gramd1c* ^−/−^ mice had modestly reduced levels of select bile acid species and elevated cortisol levels, only under low dietary cholesterol conditions. However, the vast majority of bile acids, oxysterols, and steroid hormones were unaltered in *Gramd1c* ^−/−^ mice. Bulk RNA sequencing in the liver showed that *Gramd1c* ^−/−^ mice did not exhibit alterations in sterol-sensitive genes, but instead showed altered expression of genes in major urinary protein and cytochrome P450 (CYP) families only under low dietary cholesterol conditions.

**Discussion:**

Collectively, these data indicate nominal effects of Aster-C on whole body cholesterol transport and metabolism under divergent dietary cholesterol conditions. These results strongly suggest that Aster-C alone is not sufficient to control whole body cholesterol balance, but can modestly impact circulating cortisol and bile acid levels when dietary cholesterol is limited.

## Introduction

In most mammalian cells the majority of cholesterol resides in the plasma membrane (PM), yet feedback regulation of cholesterol synthesis and metabolism of cholesterol happens at intracellular membranes of the endoplasmic reticulum (ER), mitochondria, and nucleus. Therefore, the trafficking of PM cholesterol to intracellular organelles is tightly controlled to maintain cellular homeostasis. A recently identified family of ER-resident proteins called Asters (Aster-A, -B, and -C) form membrane-membrane contact sites to facilitate the transfer cholesterol away from the PM (Sandhu et al., 2018; Naito et al., 2019; Ferrari et al., 2020; Ferrari et al., 2023; Xiao et al., 2023). Aster-B was first described as a liver X receptor (LXR)-stimulated cholesterol transporter that facilitates PM-to-ER sterol transfer for cholesterol ester (CE) storage in the adrenal gland (Sandhu et al., 2018). More recently, Aster-C was found to be a direct transcriptional target gene for the farnesoid X receptor (FXR) in the liver where it plays a role in reverse cholesterol transport (RCT) (Xiao et al., 2023). Although there is emerging evidence of functional redundancy in some tissues, all three Asters (A, B, and C) have been implicated in both intestinal and hepatic cholesterol trafficking under certain conditions of fasting and re-feeding (Ferrari et al., 2023; Xiao et al., 2023). However, the specific individual role for each Aster protein in regulating whole body cholesterol balance under conditions of excess dietary cholesterol is not well understood.

Although only 5 years have passed since their original discovery as sterol transporters by Tontonoz and colleagues (Sandhu et al., 2018), there has been rapid progress to understand the relative and tissue-specific roles each ASTER family member plays in cholesterol transport. Each Aster has a cholesterol-binding pocket that is near the N-terminal GRAM domain and are tethered to the ER via a transmembrane domain at the C terminus. The GRAM domain plays a central role in binding the plasma membrane (PM) at PM-ER contact sites where Asters can facilitate nonvesicular transfer of cholesterol to the ER for esterification or feedback regulation of *de novo* cholesterol synthesis via the sterol regulatory element-binding protein cleavage-activating protein (SCAP-SREBP) system (Sandhu et al., 2018). In addition to their roles in cellular cholesterol transport, Aster proteins have also been shown to transport carotenoid lipids (Bandara et al., 2022). Aster-C specifically has also been implicated in regulating autophagosome biogenesis and mitochondrial bioenergetics (Ng et al., 2022; Charsou et al., 2023), but how the lipid transporting function of Aster-C facilitates autophagy is still incompletely understood. One potential clue is that Aster-C has been reported to activate mTORC1 (Zhang et al., 2020), which is a master regulator of nutrient sensing and autophagy. Several recent reports have linked differential expression of *GRAMD1C* to diverse cancers including hepatocellular carcinoma, clear cell renal carcinoma, and breast cancer (Hao et al., 2019; Li et al., 2022; Fan et al., 2023; Gong et al., 2023). Although there is emerging evidence that Aster-C can facilitate sterol transport and potentially signal transduction, the relative role that Aster-C plays in tissue and whole body sterol homeostasis has yet to be defined. Here we address this gap by studying sterol balance in *Gramd1c*-deficient mice under conditions of limited *versus* excess dietary cholesterol.

## Methods

### Mouse studies

*Gramd1c* knockout mice were obtained from MMRRC UC Davis, strain 047990-UCD, *C57BL/6N-Atm1Brd Gramd1ctm1a(KOMP*)*Wtsi/JMmucd.* A breeding colony was developed at the Cleveland Clinic after the mice were genotype confirmed using a commercial partner (Transnetyx, Inc., Cordova, TN, United States). Heterozygous (*Gramd1c* ^+/−^) mice were interbred to generate littermate controls. At 8 weeks of age, female wild type (*Gramd1c* ^+/+^) or *Gramd1c* knockout mice (*Gramd1c* ^−/−^) were switched from standard rodent chow to one of two experimental synthetic diets containing low (0.02%, wt/wt) or high (0.2%, wt/wt) levels of dietary cholesterol. These experimental diets were synthesized by Envigo—Teklad Diets (Madison, WI, United States) and additional information can be found for each of these diets by referencing the following diet numbers: TD.130,104 (low cholesterol diet) and TD.160,514 (high cholesterol diet) from our previous work (Pathak et al., 2020). Mice were maintained on these diets over a period 4 consecutive weeks, and phenotyped as described below for sterol and bile acid homeostasis. In a subset of male mice, we studied liver X receptor-driven alterations in sterol balance. For LXR agonist studies, the LXR agonist GW3965 was suspended in a vehicle containing 1.0% carboxymethylcellulose (CMC) and 0.1% Tween 80. Mice were gavaged with either vehicle or 40 mg/kg GW3965 once daily for a period of seven consecutive days. All mice were maintained in an Association for the Assessment and Accreditation of Laboratory Animal Care, International-approved animal facility and all experimental protocols were approved by the Institutional Animal Care and Use Committee (IACUC) of the Cleveland Clinic (IACUC protocol # 00002499 and 00003201).

### Standardized necropsy conditions

To keep results consistent, the vast majority of experimental mice were fasted for 4 h (from 9:00 a.m. to 1:00 p.m.) prior to necropsy. At necropsy, all mice were terminally anesthetized with ketamine/xylazine (100–160 mg/kg ketamine-20–32 mg/kg xylazine), and a midline laparotomy was performed. Blood was collected by heart puncture. Following blood collection, a whole body perfusion was conducted by puncturing the inferior vena cava and slowly delivering 10 mL of saline into the heart to remove blood from tissues. Tissues were collected and immediately snap frozen in liquid nitrogen for subsequent biochemical analysis or fixed for morphological analysis.

### Quantification of total and free sterols in plasma and liver

To extract total sterols from plasma, an internal standard cocktail in 3 mL of chloroform: methanol (2:1, v/v) solution was added to 20 μL of plasma. The solution was vortexed briefly, and thereafter centrifuged at 3,000 rpm for 15 min for phase separation of the solvents. Lipids dissolved in chloroform form the supernatant layer which was transferred to a fresh glass tube. For extraction of lipids from liver tissues, an internal standard cocktail in 3 mL of chloroform: methanol (2:1, v/v) solution was added to 20–40 mg of liver tissue and mixed well. The reaction mixture was incubated overnight, and the chloroform layer containing lipid was collected in a fresh glass tube. The lipid extracts (from both plasma and liver tissue) were washed twice with water by centrifuging the solution at 3,000 rpm for 15 min. The bottom layer comprising of lipids dissolved in chloroform was collected in a fresh tube and dried under a stream of N_2_ gas. To differentiate total *versus* esterified forms of sterols, the dried lipids were resuspended in 100 μL of 0.5 M potassium hydroxide (KOH) and subjected to base hydrolysis for 1 h at 37°C. The reaction was then neutralized by adding 400 ul of 0.25 M hydrochloric acid (HCl). Thereafter, 1 mL of isopropanol:hexane:2 M acetic acid (40:10:1, v/v/v; Solution A) was added to the reaction mixture and vortexed briefly. Another 1 mL of hexane was added to the reaction mixture and the resultant solution was vortexed for ∼1 min. The solution was then centrifuged at 2,500 *g* for 5 min at 4°C, the top hexane layer collected in a fresh tube, and dried in a stream of N_2_ gas. To ensure complete drying, the lipids were put in a speed vacuum for 1 h, and later resuspended in 50 ul of Sylon™ HTP (HMDS + TMCS + Pyridine; 3:1:9) and incubated the solution at 90°C for 1 h. Thereafter the samples were transferred to glass tubes suitable for gas chromatography. The quantitation of a variety of plant and animal-derived sterols including: 7-dehydrocholesterol, brassicasterol, β-sitosterol, campesterol, cholestanone, cholesterol, coprostanol, desmosterol, ergosterol, lanosterol, lathosterol and stigmasterol were performed using isotope dilution gas chromatography-tandem mass spectrometry (GC-MS/MS) by using multiple reaction monitoring (MRM) mode. The absolute quantity of each sterol was determined using calibration curves measured for each analyte. Samples were analyzed by using the Thermo TSQ-Evo triple quadrupole in tandem with the Trace 1,310 gas chromatograph (Thermo Fisher Scientific). Chromatographic separation was achieved by using an Agilent CP-Sil 8 CB fused silica column (50 m × 0.250 mm x 0.25 µm; Agilent Technologies, Santa Clara, CA, United States) coated with 5% phenyl methylpolysiloxane. Each extract was injected (1 µL) in splitless mode for 6 min into PTV (programmable temperature vaporizer) inlet. The PTV program was as follows. Initial temp 80°C for 2 min. Evaporative stage 5°C/min to 85°C for 1 min. Transfer stage 14.5°C/min to 280°C for 5 min with cleaning stage of 14.5°C/min to 300°C for 3 min. Helium as carrier gas flow was 1 mL/min. The GC oven temperature program was as follows. The initial temperature of 150°C was held for 3 min after injection before it was increased up to 280°C at 30°C/min, followed by an increase to 295°C at 10°C/min and then held for 6 min. Argon was used a collision gas. The transfer line, and ion source temperature were set at 310°C and 275°C respectively. The mass spectrometer was tuned to an electron impact ionization energy of 70 eV in MRM mode with the following parent to daughter ion transitions: *m/z 351.3→143.2* for 7-dehydrocholesterol, *m/z 470.4→255.3* for brassicasterol, *m/z 396.4→213.2* for β-sitosterol, *m/z 402.5→219.3 D*_*6*_ *-* β-sitosterol, *m/z 382.4→213.3* for campesterol, *m/z 388.4→213.3 D*_*6*_ *—*campesterol, *m/z 386.4→231.2* for cholestanone, *m/z 368.4→213.3* for cholesterol, *m/z 375.4→213.2 D*_*7*_ *—*cholesterol, *m/z 370.4→215.3* for coprostanol, *m/z 343.3→253.3* for desmosterol, *m/z 396.65→157.1* for ergosterol, *m/z 394.4→109.2* for lanosterol, *m/z 459→213.3* for lathosterol and *m/z 484.6→169.2* for stigmasterol.

### Quantification of fecal neutral sterol excretion

Quantitative fecal excretion of both plant and animal derived sterols were analyzed by GC-MS/MS with MRM. Briefly, after being fed experimental diets for 4 weeks, mice were individually housed in a cage with a wire bottom and was allowed free access to diet and water for 3 consecutive days. After a 3 days fecal collection, the mice were weighed, and the feces were collected, dried in a 70°C vacuum oven, weighed, and crushed into a fine powder. A measured mass (50–100 mg) of feces was placed into a glass tube containing internal standard mixture. The feces were saponified and the neutral lipids were extracted into hexane, and mass analysis of the extracted neutral sterols was conducted by GC-MS/MS with MRM as described above.

### Quantification of plasma bile acid levels

Quantification of individual plasma bile acid species was conducted using a quantitative stable isotope dilution LC-MS/MS analytical method as recently described (Choucair et al., 2020). Briefly, stable isotope labeled internal standards (IS) included were: D_4_-glycolithocholic acid, D_4_-glycoursodeoxycholic acid, D_4_-glycodeoxycholic acid, D_4_-glycocholic acid, D_4_-taurolithocholic acid, D_4_-tauroursodeoxycholic acid, D_4_-taurochenodeoxycholic acid, D_4_-taurodeoxycholic acid, and D_4_-taurocholic acid; D_4_-lithocholic acid, D_4_-chenodeoxycholic acid, D_4_-deoxycholic acid, and D_4_-cholic acid, and D_4_-glycochenodeoxycholic acid. Mouse plasma samples were mixed with ice-cold methanolic IS working solution of internal standard, and were vortexed for 10 min and centrifuged (14,000 *g,* 20 min, 4°C). The supernatant was transferred to glass HPLC vials for LC/MS/MS analysis using a 4000 Q-Trap triple quadrupole tandem mass spectrometer (AB SCIEX, MA, United States) equipped with an electrospray ionization source operating in negative ion mode. Mass spectrometry parameters were as follows: ions spray voltage—4200 V, ion source heater temperature 500°C, source gas 1: 35 psi, source gas 2: 45 psi, and curtain gas 35 psi. Nitrogen gas was used for the nebulizer, curtain and collision gas. Analyses were performed using electrospray ionization in negative-ion mode with multiple reaction monitoring (MRM) of precursor and characteristic product ions specific for each monitored bile acid. The HPLC system consisted of four binary pumps (LC-20 AD), autosampler operating at 10°C (Nexera X2 SIL-30AC), controller (CBM-20A) (Shimadzu Scientific Instruments, Inc., MD, United States) and a dual column switching valve system Rheodyne (IDEX Health and Science, MA, United States). Chromatographic separations were performed on a reverse phase columns (Kinetix C18, 2.6 µm, 150 mm × 4.6 mm ID; catalog # 00F-4462-E0; Phenomenex, Torrance, CA). Mobile phase A was 1 mM ammonium acetate and 0.1% acetic acid in methnol:acetonitrile:water (1:1:3; v/v/v) and mobile phase B was 0.1% acetic acid in methanol:acetonitrile:2-propanol (4.5:4.5:1; v/v/v). Samples were injected onto columns equilibrated in 100% A, and separated using a gradient as follows: 0–2 min 0% B; 2–20 min 0%–100% B; 20–28.5 min 100% B. Flow rate was programmed as follows: 0.3 mL/min from 0–20 min, and 0.5 mL/min from 20–28 min. Samples are introduced to the mass spec for analysis from 9–28 min. To eliminate carry over, an extensive washing step alternating between mobile phase A and B was added at the end of each run as follows: 100% A for 28–35 min, then directly switched to 100% B from 36–46 min, and equilibration step of 100% A from 46–60 min. To increase sample throughput 2-fold, a dual chromatographic system was used. At 28 min of the gradient on the first column, the next sample was injected into a second column; thus, during the first column washing and equilibration, the second column is used for BAs separation and diversion to the mass spectrometer for analysis. Calibration curves were built by fitting each analyte concentration (10 different points) to peak area ratios (analyte/internal standard). The limit of detection (LOD) was defined as the lowest concentration of analyte in sample matrix (e.g., serum) that generated a signal-to-noise ratio of ≥3. The limit of quantification (LOQ) was defined as the lowest concentration of analyte in sample matrix that generated a signal-to-noise ratio of ≥10. Recovery was tested by comparing area of deuterated standards added to pooled human serum (mix of ≥10 equal serum aliquots from healthy normal subjects) *versus* methanol, and calculated according to the following formula: % recovery = (average area spiked in serum pool/average area spiked in methanol) x100.

### Quantification of plasma steroid hormone levels

A targeted steroid hormone panel from plasma including extraction was performed by the West Coast Metabolomics Center at the University of California—Davis (Director—Dr. Oliver Fiehn). The extraction protocol was adapted from Pedersen and Newman. 2018. Plasma sample was vortexed and 50 µL of Surrogate Standards of the 3 lipid classes was added along with CDU and vortexed again for 30sec. Samples were centrifuged at 6°C for 5 min at 15,000 g and the supernatant (∼240 µL) was transferred into new Eppendorf tube/filter plate Spin Filter (0.1 um) and stored in −20°C until analysis. The samples were run using Thermo Scientific Vanquish Horizon UPLC/SciEx QTrap 6,500+.

### Quantification of liver oxysterol levels

Lipids were extracted by addition of 2 mL hexane/isopropanol (3:2, v/v) or chloroform/methanol (2:1, v/v) from the cell monolayers and liver tissue, respectively. 24–25- and 27- hydroxy cholesterol levels were quantified by isotope dilution mass spectrometry as previously described (Dzeletovic et al., 1995). Oxysterol levels were normalized for the total protein content measured using the RC DC™ Protein Assay (BioRad Laboratories Inc., United States) in liver tissue after digestion with NaOH (0.25 mol/L).

### Liver targeted metabolomics

The AbsoluteIDQ^®^ p400 HR kit from Biocrates Life Sciences AG was used to obtain targeted quantitative metabolomics data as we have previously described (Osborn et al., 2019). Liver tissue was collected at time of necropsy and samples were prepared according to manufacturer protocol. Briefly, at least 30 mg of each tissue sample was cut and weighed, and kept frozen throughout this protocol. The tissue samples were homogenized and centrifuged at 10,000 × g for 5 min. The supernatant was then collected, 10 μL of which per sample was loaded onto a 96-well plate containing stable isotope-labeled standards, and processed according to manufacturer protocol. The LCMS analysis was done using specific parameters (both Tune and LCMS methods) per kit manufacturer’s recommendations. The assay was performed on a Q-Exactive HF (operated only in positive ESI mode) coupled with a Vanquish UHPLC + focused liquid chromatography as detailed per assay instructions. The manufacturer-provided software, MetIDQ (Biocrates, Life Science AG), was used to provide the peak identification.

### Lipoprotein cholesterol distribution by size-exclusion chromatography

Plasma (40–50 mL) was diluted to a total volume of 500 mL in PBS and loaded into an Akta Pure 25L liquid chromatography system. Samples were passed through a Superose 6 Increase column (Cytiva) at a flow rate of 0.75 mL/min in PBS. Fractions were collected (0.5 mL/fraction) and assayed for total cholesterol content by enzymatic assay (Fujifilm; catalog # 999–02601). Independent runs were performed for each mouse, and cholesterol distribution in very low density (VLDL), low density (LDL), and high density (HDL) lipoproteins was calculated based on the percent distribution in each fraction compared to the total plasma cholesterol measured by GC-MS/MS.

### Real-Time PCR and bulk RNA sequencing analysis of gene expression

Tissue RNA extraction and quantitative polymerase chain reaction (qPCR) analyses of relative mRNA abundance were conducted as previously described (Helsley et al., 2019; Pathak et al., 2020). mRNA expression levels were calculated based on the ΔΔ-CT method using Cyclophilin A as a housekeeping gene. qPCR was conducted using SYBR Fast reagents (AB#4385612) on the Applied Biosystems 7,500 Real-Time PCR System. Primers used for qPCR are *CycloA* F - GCG​GCA​GGT​CCA​TCT​ACG, *CycloA* R - GCC​ATC​CAG​CCA​TTC​AGT​C, *Gramd1a* F - CAT​GCA​CAC​CTC​AGG​TTC​CC, *Gramd1a* R - ACG​ATG​AGG​ACA​ATG​CTG​ATG, *Gramd1b* F - GCT​GGT​TAT​CAG​CTG​TGT​TCT​G, *Gramd1b* R - GTG​AGG​GTC​TGG​GTG​GTG​TA, *Gramd1c* F -CAG​TTA​TGA​CAC​CGC​CCT​TAT *Gramd1c* R - CTG​GGT​AGC​GTG​TTC​TAT​CTT​T. RNA extraction, library preparation, sequencing and analysis was conducted at Azenta Life Sciences (South Plainfield, NJ, United States) as follows: Extraction: Total RNA was extracted using Qiagen Rneasy Plus Universal Mini kit following manufacturer’s instructions (Qiagen, Hilden, Germany). Library Preparation with PolyA selection and Illumina Sequencing: RNA samples were quantified using Qubit 2.0 Fluorometer (Life Technologies, Carlsbad, CA, United States) and RNA integrity was checked using Agilent TapeStation 4,200 (Agilent Technologies, Palo Alto, CA, United States). RNA sequencing libraries were prepared using the NEBNext Ultra RNA Library Prep Kit for Illumina using manufacturer’s instructions (NEB, Ipswich, MA, United States). Briefly, mRNAs were initially enriched with Oligod(T) beads. Enriched mRNAs were fragmented for 15 min at 94°C. First strand and second strand cDNA were subsequently synthesized. cDNA fragments were end repaired and adenylated at 3′ends, and universal adapters were ligated to cDNA fragments, followed by index addition and library enrichment by PCR with limited cycles. The sequencing library was validated on the Agilent TapeStation (Agilent Technologies, Palo Alto, CA, United States), and quantified by using Qubit 2.0 Fluorometer (Invitrogen, Carlsbad, CA) as well as by quantitative PCR (KAPA Biosystems, Wilmington, MA, United States). The sequencing libraries were multiplexed and clustered onto a flowcell on the Illumina NovaSeq instrument according to manufacturer’s instructions. The samples were sequenced using a 2 × 150 bp Paired End (PE) configuration. Image analysis and base calling were conducted by the NovaSeq Control Software (NCS). Raw sequence data (.bcl files) generated from Illumina NovaSeq was converted into fastq files and de-multiplexed using Illumina bcl2fastq 2.20 software. One mis-match was allowed for index sequence identification. Transcript per million (TPM) values for each differentially expressed gene were plotted using Prism Software and the statistical analysis was done using JMP software.

### Immunoblotting

Whole liver homogenates were made from tissues in a modified RIPA buffer (Abcam #156034) as previously described (Helsley et al., 2019; Pathak et al., 2020), and protein was quantified using the bicinchoninic (BCA) assay (Pierce). Proteins were separated by 4%–12% SDS-PAGE, transferred to polyvinylidene difluoride (PVDF) membranes (ThermoSci #88518), and proteins were detected after incubation with an antibody recognizing mouse ASTER-C (generated here) or β-actin (Cell Signaling Technologies product # 4970S). Given the paucity of available antibodies, a custom rabbit polyclonal antibody was generated in collaboration with Thermofisher. The antigen used was the N-terminus 34 kDa mouse Aster-C protein (Uniprot Q8CI52; amino acids 1–300). A 72 days injection protocol with a primary immunization and three subsequent boosters was followed, and the terminal 72 days crude anti-sera sample (50 mL) was affinity purified (expressed protein conjugated to the affinity column) by ThermoFisher. Affinity-purified antibodies were eluted using a step-wise pH gradient and collected in neutralizing buffer. Purified antibodies were then concentrated and final concentration is measured by BCA. Membrane was blocked with 5% milk in Tris buffered saline containing 0.2% Tween 20.1:500 dilution of this Gramd1c antibody in Blocking buffer was used for Western blotting and the bands were detected using an anti-rabbit-HRP conjugated secondary antibody.

### Cell culture and generation of *Gramd1c-Deficient RAW264.7 cells*

Mycoplasma-tested RAW264.7 macrophage cells were cultured under standard conditions in Dulbecco-modified Eagle’s minimum essential medium (D-MEM) (GIBCO, Life Technologies, Carlsbad, CA) supplemented with 10% fetal bovine serum (FBS, GIBCO), 1% l-glutamine, 1% penicillin-streptomycin and 1% nonessential amino acids in a 5% CO2-humidified chamber at 37°C.

CRISPR-Cas9 genome editing was accomplished using methods previously described (Ran et al., 2013). Gramd1c sgRNAs were designed by an online tool (https://www.benchling.com/) and cloned into the Lenti-CRISPER v2 vector (Addgene (Ran et al., 2013) with D10A nickase version of Cas9 (Cas9n)). *Gramd1c KO* cell lines were generated following lentiviral transduction of the Lenti-CRISPER v2-Cas9 D10A- Gramd1c sgRNA in RAW264.7cells. *Gramd1c KO* cells were validated by analyzing the expression of GRAMD1c by Western blot. Primers used for gene editing were: *GRAMD1c*-E5-Nick-5F: 5′-CAC​CGC​AGA​GCA​CCC​TCC​AAG​TCA​C-3′, *GRAMD1c*-E5-Nick-5R: 5′-AAA​CGT​GAC​TTG​GAG​GGT​GCT​CTG​C-3′, *GRAMD1c*-E5-Nick-3F: 5′- CAC​CGC​TCT​TTT​CTG​AAG​CTA​GCG​A-3′, and *GRAMD1c*-E5-Nick-3R: 5′- AAA​CTC​GCT​AGC​TTC​AGA​AAA​GAG​C-3’.

### cDNA cloning and expression of GRAMD1c

*Gramd1C* construct was cloned by double-stranded gene fragment synthesis (IDT, Coralville, IA) and assembled using NEBuilder^®^ HiFi DNA Assembly (NEB, Ipswich, MA). The double-stranded gene fragment was assembled in a pLENTI-EF1alpha-3XFLAG vector backbone. pLENTI-EF1alpha-3XFLAG-Gramd1c was expressed in RAW264.7 cells by transient transfection using Lipofectamine 3,000. Cells were harvested 72 h after transfection in RIPA lysis buffer supplemented with complete protease inhibitors (Roche Applied Science). Lysates were cleared by centrifugation at 16,000 × *g* for 20 min, and Western blots were performed as described above as well as in the online data supplement.

### Statistical analysis

All data were analyzed using one-way analysis of variance (ANOVA) where appropriate, followed by either a Tukey’s or Student’s t tests for *post hoc* analysis. Differences were considered significant at *p* < 0.05. All mouse data analyses were performed using Graphpad Prism 6 (La Jolla, CA, United States) software or JMP version 17 (SAS Institute, Cary, NC, United States) software.

## Results

### Global deletion of *Gramd1c* does not alter circulating sterol levels

**FIGURE 1:**
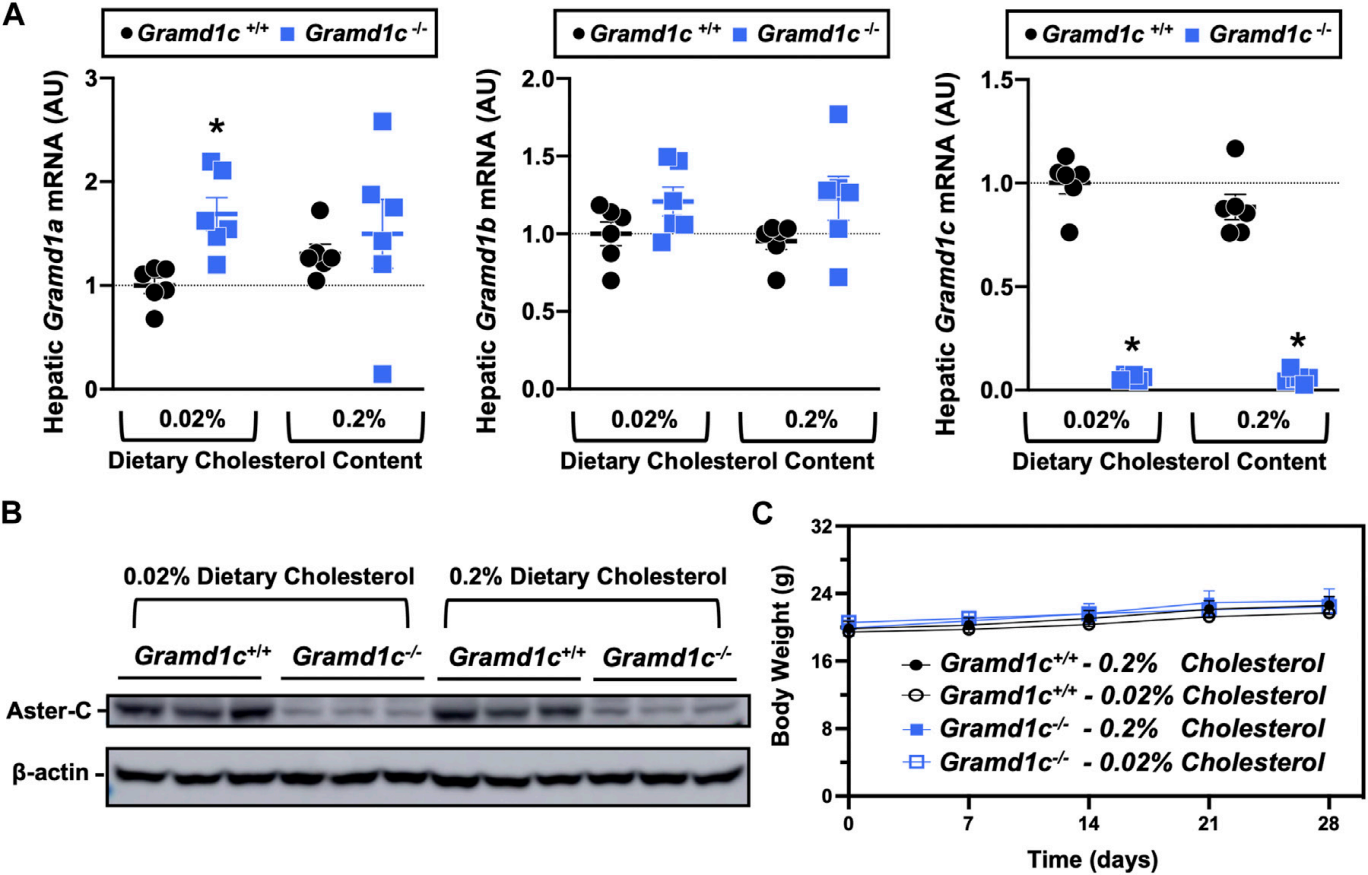
Selective loss of *Gramd1c* does not alter body weight. At 8 weeks of age, female wild type (*Gramd1c* ^+/+^) or *Gramd1c* knockout mice (*Gramd1c* ^−/−^) were switched from standard rodent chow to one of two experimental synthetic diets containing low (0.02%, wt/wt) or high (0.2%, wt/wt) levels of dietary cholesterol. Mice were maintained on these diets over a 4-week period of study. **(A)** The expression of *Gramd1a*, *Gramd1b*, and *Gramd1c* in the liver was quantified via qPCR. **(B)** Western blot in liver lysates from n = 3 mice per group. **(C)** Body weight curves over the 4-week feeding period. Data are presented as mean ± SEM from n = three to six mice per group. There were no statistically significant differences between *Gramd1c* ^+/+^ and *Gramd1c* ^−/−^ mice on either the low or high cholesterol diets.

**FIGURE 2:**
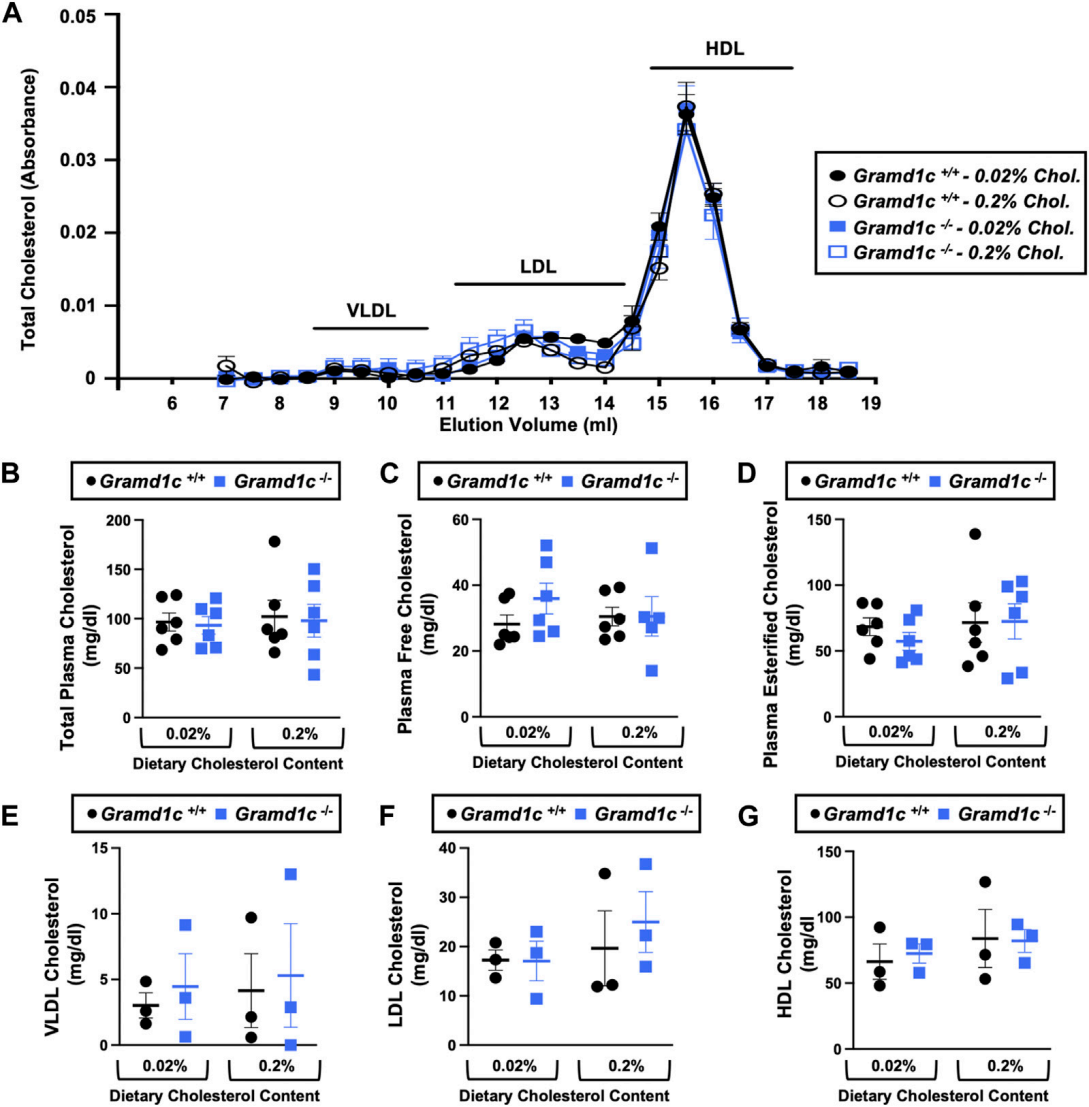
Global deletion of *Gramd1c* does not significantly alter circulating lipoprotein cholesterol levels. At 8 weeks of age, female wild type (*Gramd1c* ^+/+^) or *Gramd1c* knockout mice (*Gramd1c* ^−/−^) were switched from standard rodent chow to one of two experimental synthetic diets containing low (0.02%, wt/wt) or high (0.2%, wt/wt) levels of dietary cholesterol. Mice were maintained on these diets over a 4-week period of study. **(A)** Plasma was subjected to size exclusion chromatography to examine total cholesterol distribution across lipoprotein fractions. **(B–D)** Total plasma cholesterol **(B)**, total plasma free cholesterol **(C)**, and total plasma esterified cholesterol **(D)** levels were determined by gas chromatography-tandem mass spectrometry (GC-MS/MS). **(E–G)** Plasma very low-density lipoprotein (VLDL), low-density lipoprotein (LDL), and high-density lipoprotein (HDL) cholesterol levels. Data are presented as mean ± SEM from n = three to six mice per group. There were no statistically significant differences between *Gramd1c* ^+/+^ and *Gramd1c* ^−/−^ mice on either the low or high cholesterol diets.

**FIGURE 3:**
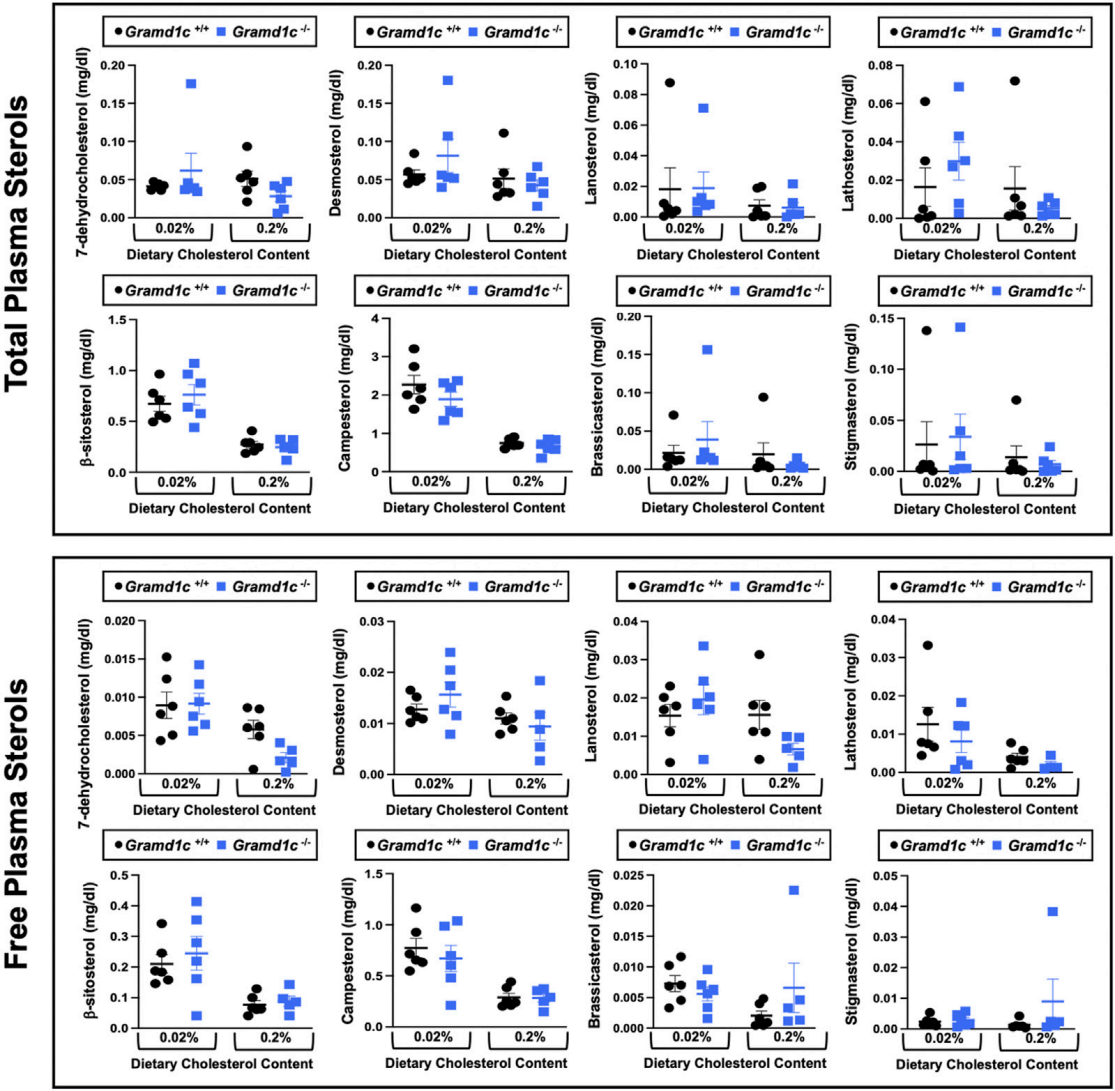
Global deletion of *Gramd1c* does not significantly alter circulating levels of various sterols. At 8 weeks of age, female wild type (*Gramd1c* ^+/+^) or *Gramd1c* knockout mice (*Gramd1c* ^−/−^) were switched from standard rodent chow to one of two experimental synthetic diets containing low (0.02%, wt/wt) or high (0.2%, wt/wt) levels of dietary cholesterol. Mice were maintained on these diets over a 4-week period of study. The total and free levels of a variety of sterols in the liver were quantified by gas chromatography tandem mass spectrometry (GC-MS/MS) in samples with or without alkaline hydrolysis. Data are presented as mean ± SEM from n = five to six mice per group. There were no statistically significant differences between *Gramd1c* ^+/+^ and *Gramd1c* ^−/−^ mice on either the low or high cholesterol diets.

To examine whether Aster-C can alter systemic cholesterol metabolism under normal physiologic conditions, we fed young wild type (*Gramd1c* ^+/+^) or global *Gramd1c* knockout mice (*Gramd1c* ^−/−^) one of two experimental diets containing low (0.02%, wt/wt) or high (0.2%, wt/wt) levels of dietary cholesterol for a period up to 4 weeks. Deletion of *Gramd1c* was confirmed in the liver, where both messenger RNA (Figure 1A) and protein (Figure 1B) were significantly reduced in *Gramd1c* ^−/−^ mice compared to littermate controls (*Gramd1c* ^+/+^). *Gramd1c* ^−/−^ mice were born at normal Mendelian ratios and were indistinguishable from wild type littermates. Irrespective of dietary cholesterol level, *Gramd1c* ^−/−^ mice maintained similar body weights compared to wild type mice throughout the study (Figure 1C). When we examined either the total, unesterified (free), or esterified levels of cholesterol in the plasma, *Gramd1c* ^−/−^ mice were not significantly different when compared to wild type controls (Figures 2B–D). Likewise, the cholesterol levels in very low-density (VLDL), low-density (LDL), or high-density lipoproteins (HDL) were similar when comparing *Gramd1c* ^+/+^ to *Gramd1c* ^−/−^ mice (Figures 2A,E,G). Given plasma cholesterol levels were unchanged in *Gramd1c* ^−/−^ mice (Figure 2), we wanted to look more broadly at other potential sterol substrates including several phytosterols and biosynthetic intermediates generated during the *de novo* synthesis of cholesterol. It is important to note that *Gramd1c* ^−/−^ mice had normal circulating levels of 7-dehydrocholesterol, desmosterol, lanosterol, lathosterol, β-sitosterol, campesterol, brassicasterol, and stigmasterol (Figure 3). These data show that Aster-C alone does not play a quantitatively important role in determining the circulating levels of cholesterol or other diverse sterols of plant or mammalian origin under conditions of low and excess dietary cholesterol.

### Aster-C modestly shapes the hepatic lipidome

**FIGURE 4:**
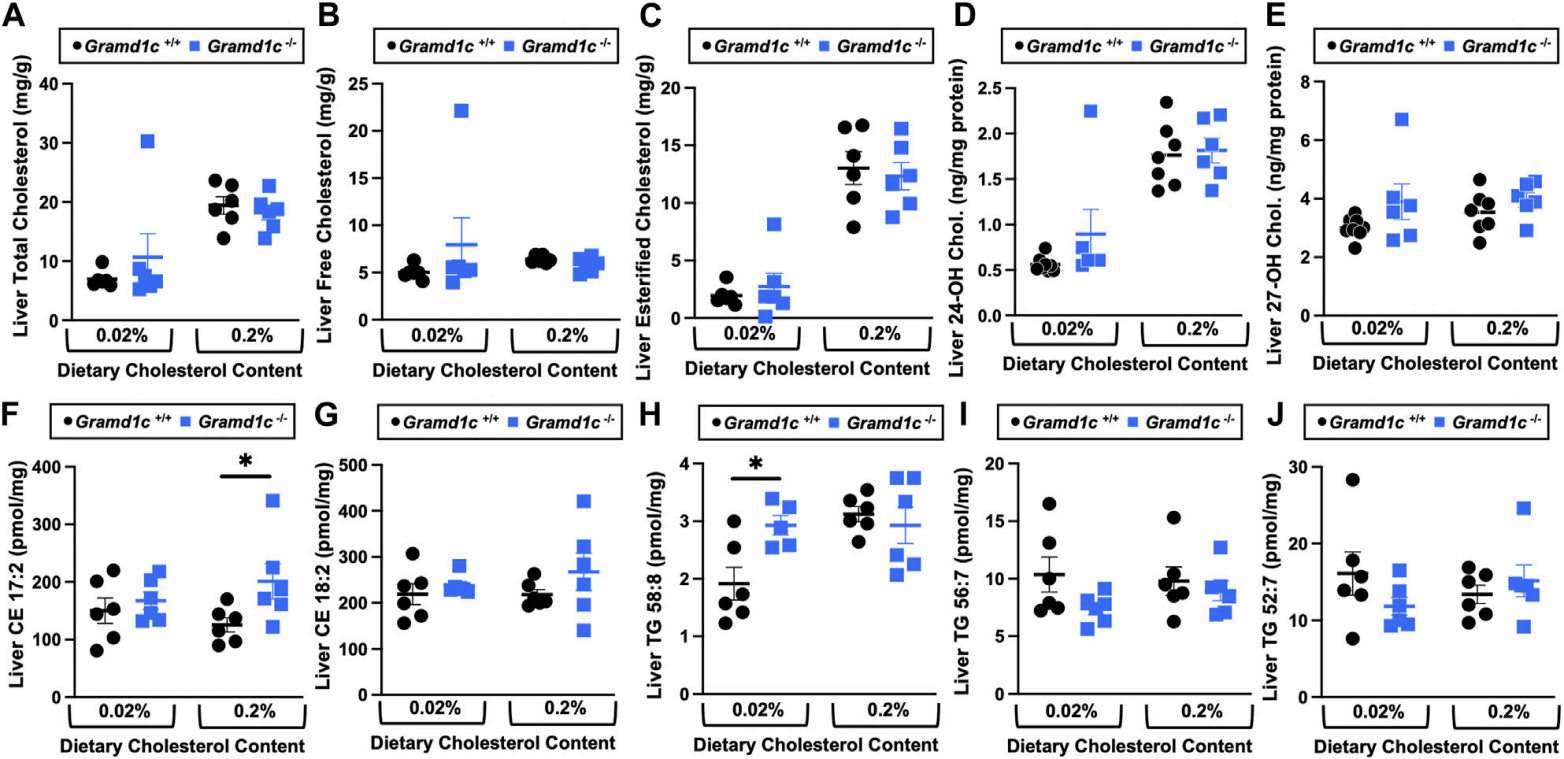
The hepatic lipidome is modestly impacted by global deletion of *Gramd1c*. At 8 weeks of age, female wild type (*Gramd1c* ^+/+^) or *Gramd1c* knockout mice (*Gramd1c* ^−/−^) were switched from standard rodent chow to one of two experimental synthetic diets containing low (0.02%, wt/wt) or high (0.2%, wt/wt) levels of dietary cholesterol. Mice were maintained on these diets over a 4-week period of study. A variety of lipids were quantified by targeted mass spectrometry assays including **(A)** total cholesterol, **(B)** free cholesterol, **(C)** total esterified cholesterol, **(D)** 24-hydoxycholesterol (24-OH Chol.), **(E)** 27-hydroxycholesterol (27-OH Chol.) **(F)** 17:2 cholesteryl ester (CE 17:2), **(G)** 18:2 cholesteryl ester (CE 18:2), **(H)** 58:8 triacylglycerol (TG 58:8), **(I)** 56:7 triacylglycerol (TG 56:7), and **(J)** 52:7 triacylglycerol (TG 52:7). Data are presented as mean ± SEM from n = 6 mice per group. * = significantly different (*p* ≤ 0.05) when comparing *Gramd1c* ^+/+^ and *Gramd1c* ^−/−^ mice within each diet group.

**FIGURE 5:**
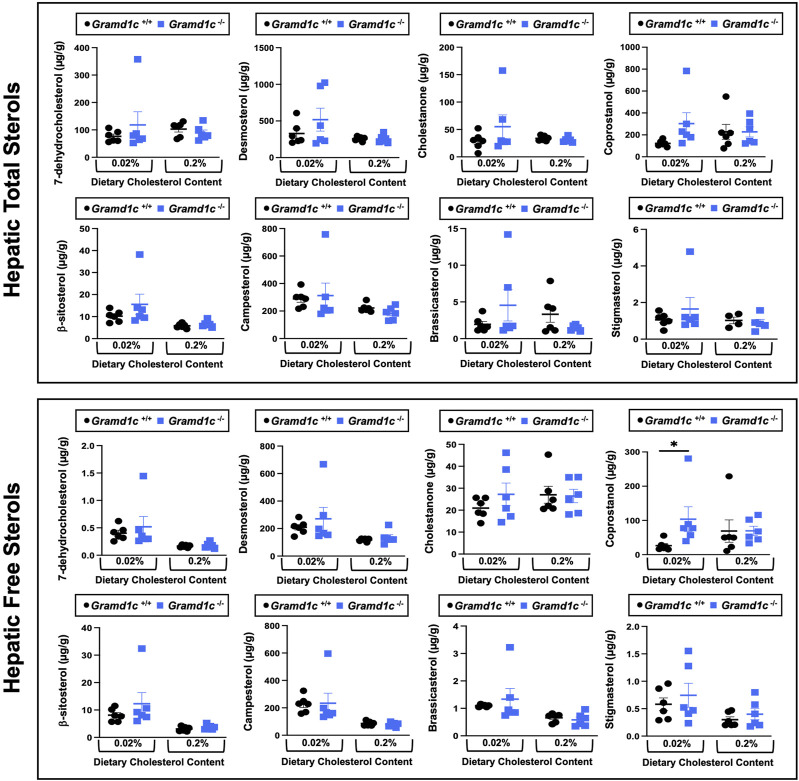
Global deletion of *Gramd1c* does not significantly alter levels of various sterols in the liver. At 8 weeks of age, female wild type (*Gramd1c* ^+/+^) or *Gramd1c* knockout mice (*Gramd1c* ^−/−^) were switched from standard rodent chow to one of two experimental synthetic diets containing low (0.02%, wt/wt) or high (0.2%, wt/wt) levels of dietary cholesterol. Mice were maintained on these diets over a 4-week period of study. The total and free levels of a variety of sterols in the liver were quantified by gas chromatography tandem mass spectrometry (GC-MS/MS) in samples with or without alkaline hydrolysis. Data are presented as mean ± SEM from n = 6 mice per group. There were no statistically significant differences between *Gramd1c* ^+/+^ and *Gramd1c* ^−/−^ mice on either the low or high cholesterol diets.

We next turned our attention to test whether Aster-C plays a role in sterol homeostasis in the liver, given the central role the liver plays in whole body cholesterol balance. Here, we applied several complimentary and broad targeted lipidomic methods to quantify diverse sterols, but also major classes of glycerophospholipids and neutral lipids. Similar to findings in plasma (Figure 2), compared to wild type mice, *Gramd1c* ^−/−^ mice have unaltered levels of total, free, and esterified cholesterol in the liver (Figures 4A–C). *Gramd1c* ^+/+^ and *Gramd1c* ^−/−^ mice also had similar hepatic levels of 7-dehydrocholesterol, desmosterol, lanosterol, lathosterol, β-sitosterol, campesterol, brassicasterol, and stigmasterol (Figure 5). We next examined the hepatic levels of the major oxysterol species that are generated from cholesterol substrate (24-hydroxycholesterol and 27-hydroxycholesterol), and found similar levels in *Gramd1c* ^+/+^ and *Gramd1c* ^−/−^ mice (Figures 4D,E). Finally, we performed a broad lipidomic approach to examine a variety of glycerophospholipid and neutral lipids in the liver. *Gramd1c* ^+/+^ and *Gramd1c* ^−/−^ mice had similar levels of all molecular species of glycerophospholipids detected (data not shown). However, this broader method was able to uncover a modest increase in cholesterol esters containing a 17:2 fatty acyl chain in high cholesterol diet-fed mice (Figure 4F). Yet, all other molecular species of cholesteryl esters were unchanged in *Gramd1c* ^−/−^ mice (Figure 4G). Furthermore, under low dietary cholesterol conditions, one specific molecular species of triacylglycerol (TG 58:8) was increased in *Gramd1c* ^−/−^ mice (Figure 4H), but all other detected species of triacylglycerol were similar to wild to mice (Figures 4I,J).

### Aster-C shapes bile acid homeostasis but does not impact fecal sterol loss

**FIGURE 6:**
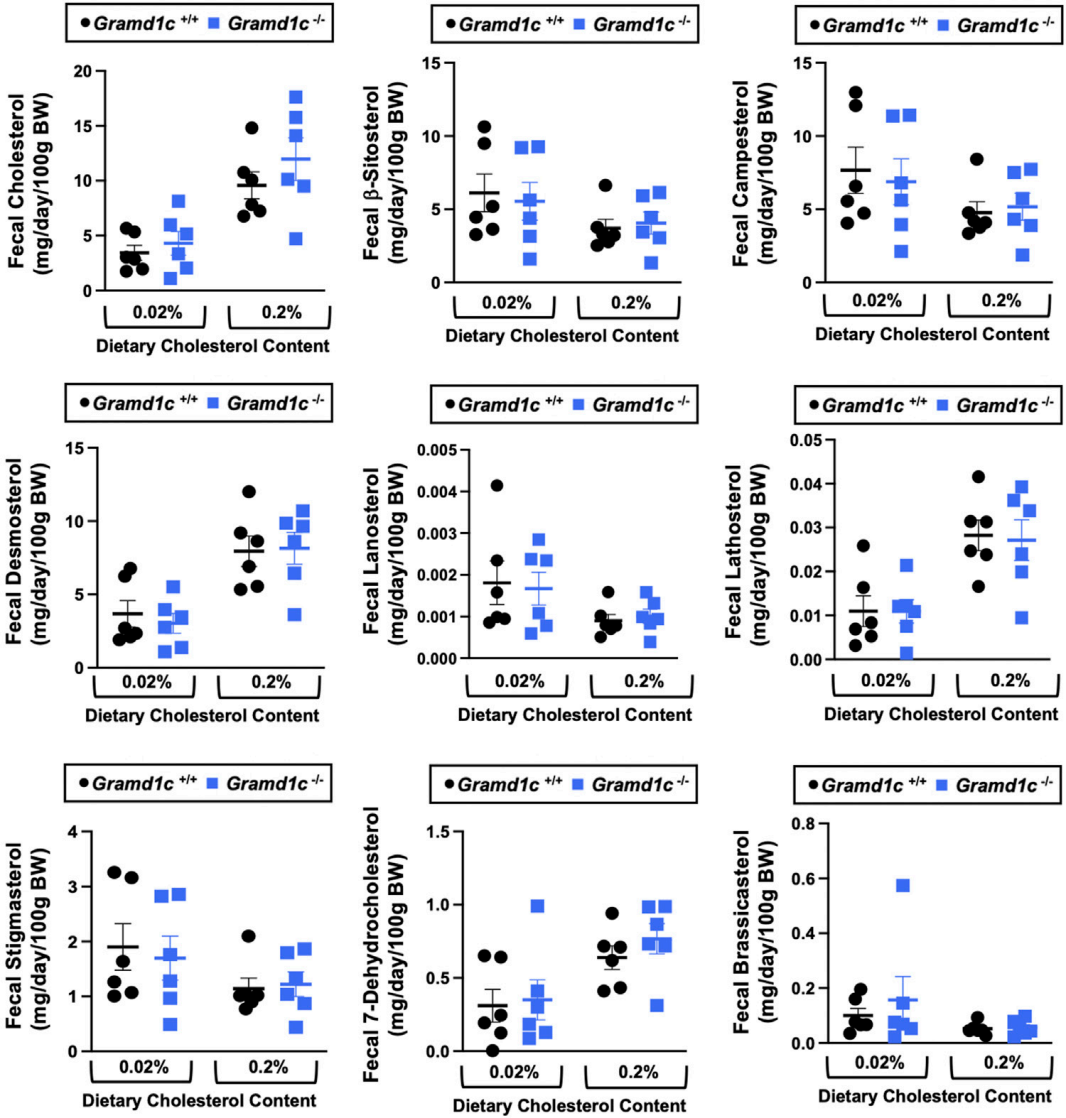
Global deletion of *Gramd1c* does not significantly alter fecal sterol loss. At 8 weeks of age, female wild type (*Gramd1c* ^+/+^) or *Gramd1c* knockout mice (*Gramd1c* ^−/−^) were switched from standard rodent chow to one of two experimental synthetic diets containing low (0.02%, wt/wt) or high (0.2%, wt/wt) levels of dietary cholesterol. Mice were maintained on these diets over a 4-week period of study, and feces were quantitatively collected over the final 72 h for fecal sterol analyses via gas chromatography tandem mass spectrometry (GC-MS/MS). Data are presented as mean ± SEM from n = 6 mice per group. There were no statistically significant differences between *Gramd1c* ^+/+^ and *Gramd1c* ^−/−^ mice on either the low or high cholesterol diets.

**FIGURE 7:**
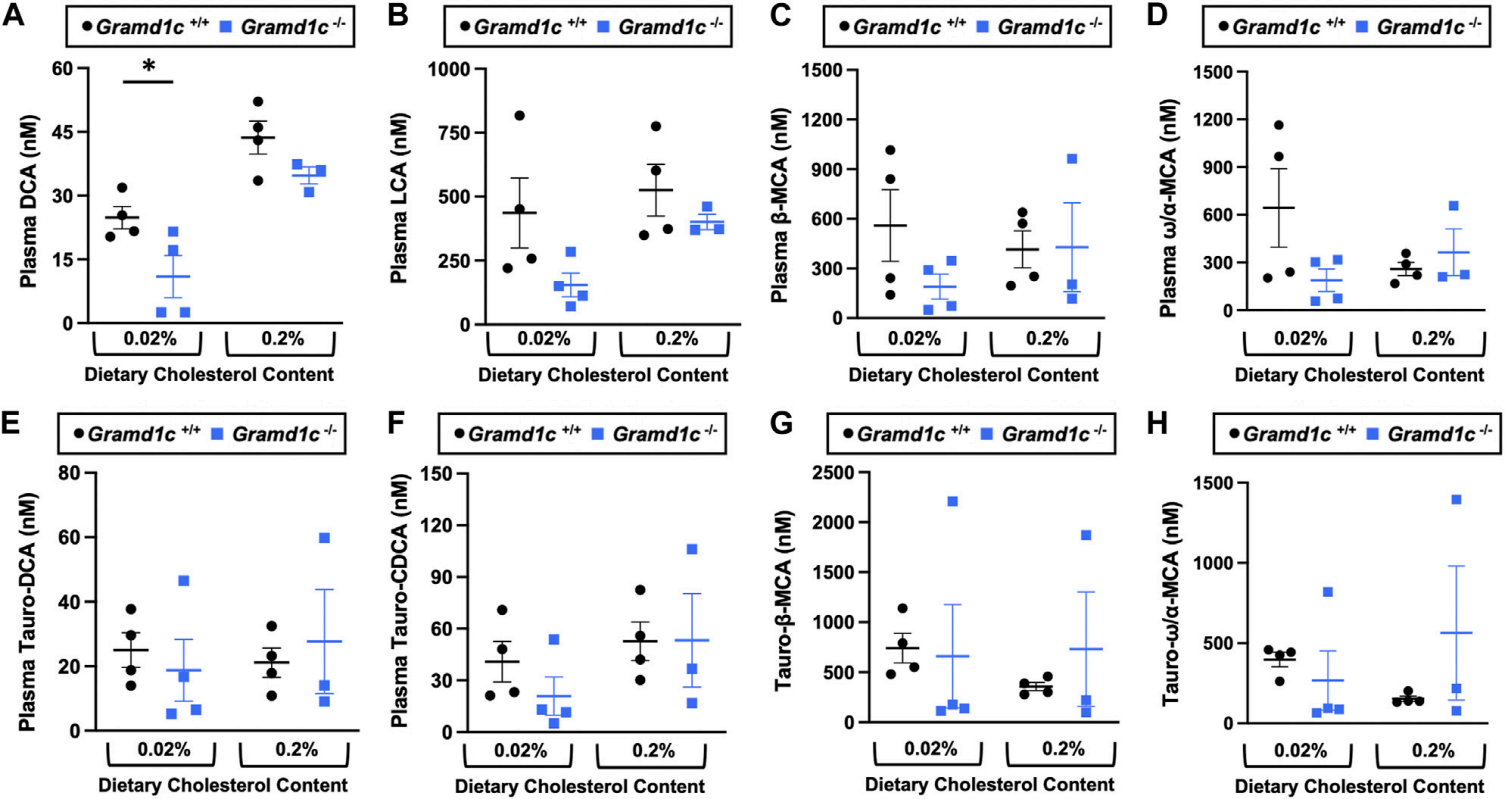
The impact of *Gramd1c* deficiency on plasma bile acids. At 8 weeks of age, female wild type (*Gramd1c* ^+/+^) or *Gramd1c* knockout mice (*Gramd1c* ^−/−^) were switched from standard rodent chow to one of two experimental synthetic diets containing low (0.02%, wt/wt) or high (0.2%, wt/wt) levels of dietary cholesterol. Mice were maintained on these diets over a 4-week period of study. The levels of various bile acids including **(A)** deoxycholic acid (DCA), **(B)** lithocholic acid (LCA), **(C)** β-muricholic acid (β-MCA), **(D)** ω/α-muricholic acid (ω/α-MCA), **(E)** taurodeoxycholic acid (Taura-DCA), **(F)** taurochenodeoxycholic acid (Tauro-CDCA), **(G)** tauro-β-muricholic acid (Taura-β-MCA), and **(H)** tauro-ω/α-muricholic acid (Tauro-ω/α-MCA) were quantified by liquid chromatography-tandem mass spectrometry (LC-MS/MS). Data are presented as mean ± SEM from n = three to four mice per group. * = significantly different (*p* ≤ 0.05) when comparing *Gramd1c* ^+/+^ and *Gramd1c* ^−/−^ mice within each diet group.

**FIGURE 8:**
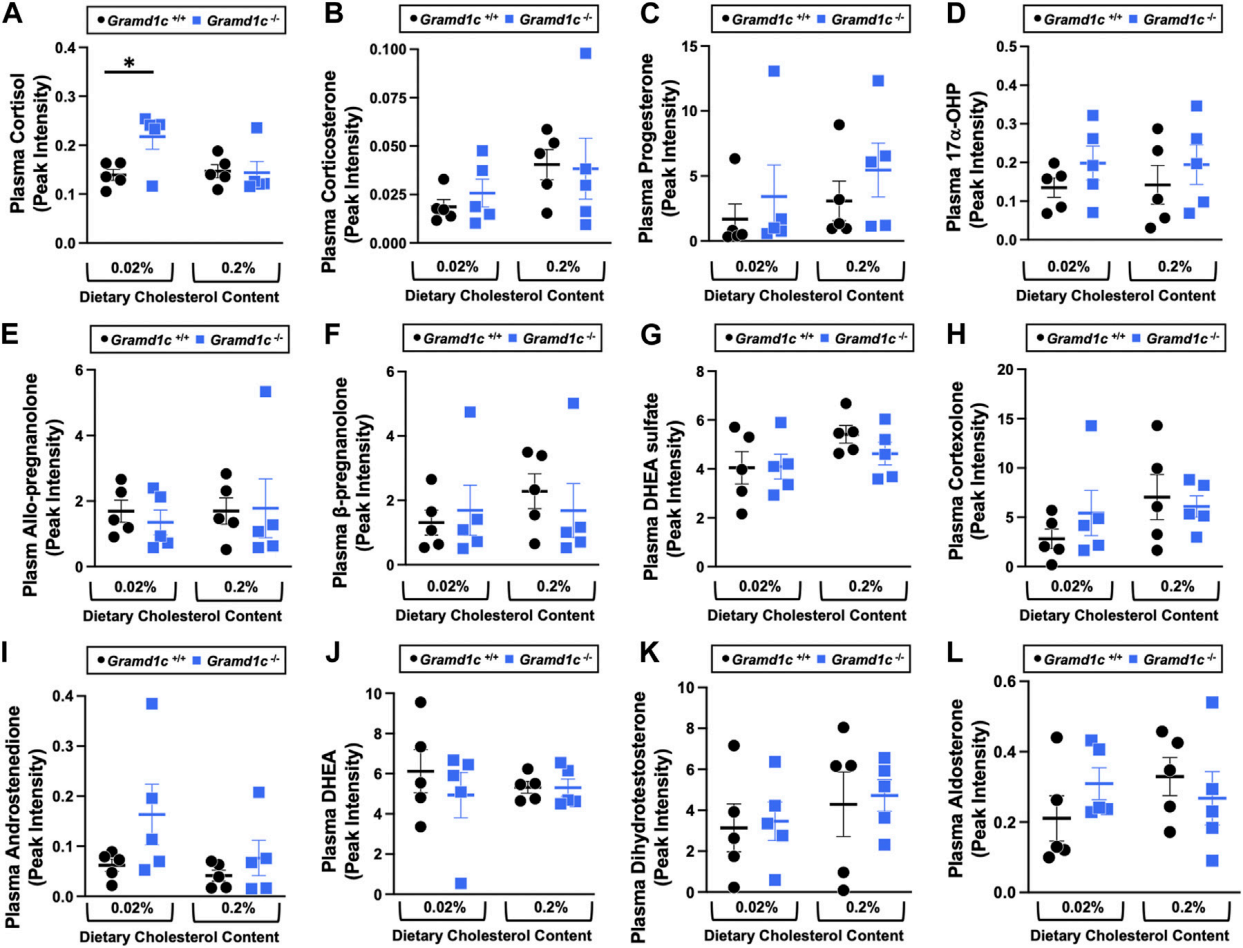
The impact of *Gramd1c* deficiency on plasma steroid hormones. At 8 weeks of age, female wild type (*Gramd1c* ^+/+^) or *Gramd1c* knockout mice (*Gramd1c* ^−/−^) were switched from standard rodent chow to one of two experimental synthetic diets containing low (0.02%, wt/wt) or high (0.2%, wt/wt) levels of dietary cholesterol. Mice were maintained on these diets over a 4-week period of study. The plasma levels of various steroid hormones including **(A)** cortisol, **(B)** corticosterone, **(C)** progesterone, **(D)** 17-α-hydroxyprogesterone, **(E)** allo-pregnanolone, **(F)** β-pregnanolone, **(G)** dehydroepiandrosterone (DHEA) sulfate, **(H)** cortexolone, **(I)** androstenedione, **(J)** dehydroepiandrosterone (DHEA), **(K)** dihydrotestosterone, and **(L)** aldosterone were measured semi-quantitatively by liquid chromatography-tandem mass spectrometry (LC-MS/MS). Data are presented as mean ± SEM from n = 5 mice per group.). * = significantly different (*p* ≤ 0.05) when comparing *Gramd1c* ^+/+^ and *Gramd1c* ^−/−^ mice within the low cholesterol diet.

A recent report by Xiao and colleagues reported that mice lacking both Aster-A and Aster-C selectively in hepatocytes have impaired reverse cholesterol transport (RCT) (Xiao et al., 2023). This important work showed that Aster-A/Aster-C double knockout mice have reduced movement of cholesterol radiotracer (^14^C-cholesterol) into the feces when either derived from LDL or HDL sources (Xiao et al., 2023). However, they did not test whether Aster-C alone can impact the mass amount of cholesterol excreted in the feces. When we examined the quantitative fecal excretion of cholesterol in global *Gramd1c* ^−/−^ mice fed low or high cholesterol, there was no significant difference when compared to wild type littermates (Figure 6). Likewise, the fecal excretion of other phytosterols and sterol intermediates such as 7-dehydrocholesterol, desmosterol, lanosterol, lathosterol, β-sitosterol, campesterol, brassicasterol, and stigmasterol were similar in *Gramd1c* ^+/+^ and *Gramd1c* ^−/−^ mice (Figure 6). Another way the liver can facilitate RCT is via the enzymatic conversion of cholesterol into bile acids, which can further contribute to removal of cholesterol from the body. Although the majority of plasma bile acid species were unchanged in *Gramd1c* ^−/−^ mice, levels of deoxycholic acid were significantly reduced in *Gramd1c* ^−/−^ mice fed a low cholesterol diet (Figure 7). Given the clear role that Aster-B plays in adrenal gland cholesterol homeostasis (Sandhu et al., 2018), we also wanted to understand whether Aster-C may play a role in cholesterol-derived hormone production. Only under low dietary cholesterol conditions, *Gramd1c* ^−/−^ mice have elevated plasma cortisol compared to wild type controls (Figure 8A). However, all other steroid hormones measured including corticosterone, progesterone, 17-α-hydroxyprogesterone, allo-pregnanolone, β-pregnanolone, dehydroepiandrosterone (DHEA) sulfate, cortexolone, androstenedione, free DHEA, dihydrotestosterone, and aldosterone were unaltered in *Gramd1c−/−* mice (Figures 8B–L).

### *Gramd1c*-deficient mice show diet-dependent alterations in hepatic gene expression

**FIGURE 9:**
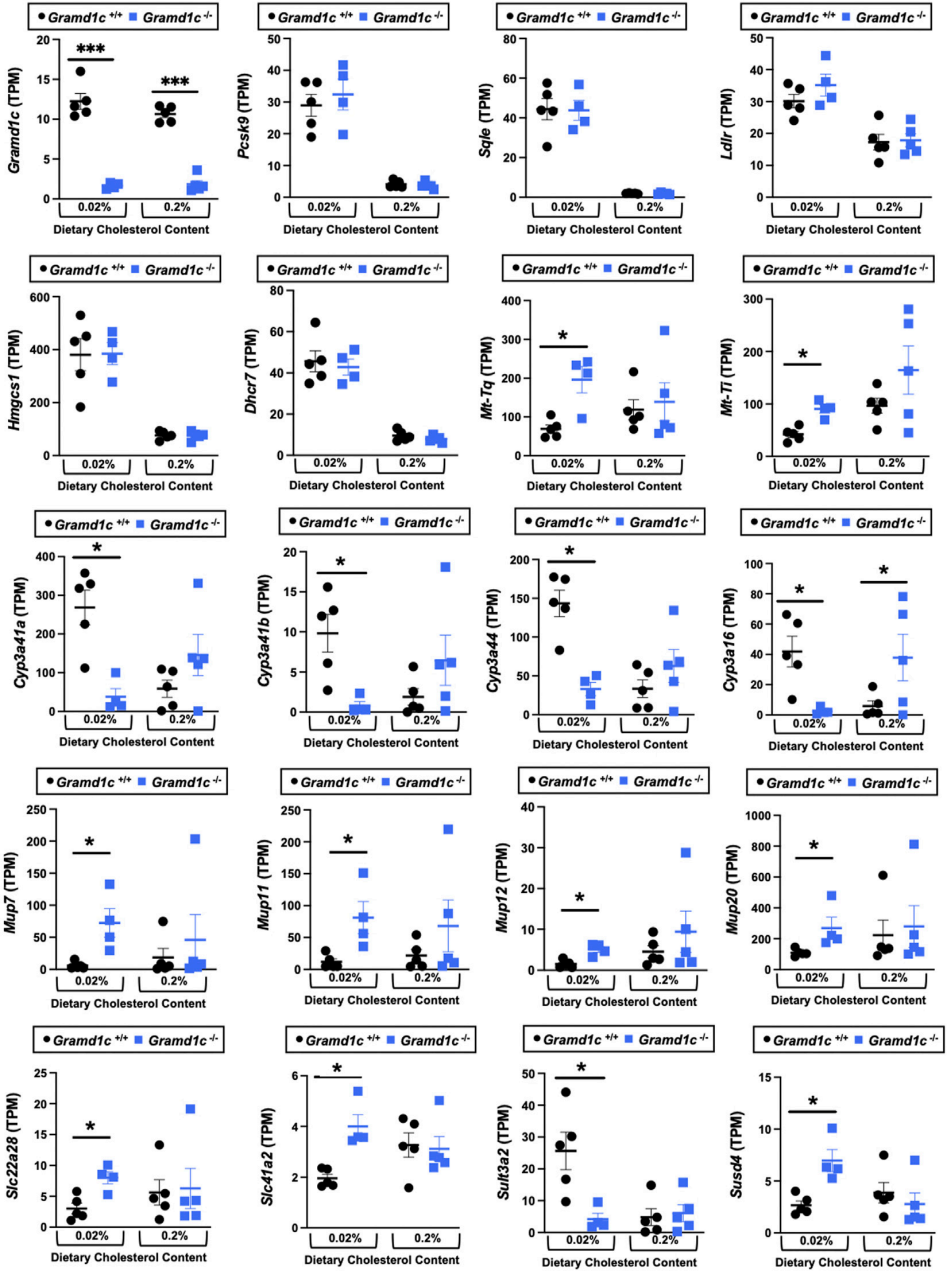
Bulk RNA sequencing in the liver of *Gramd1c−/−* mice identifies alterations in a small subset of genes. At 8 weeks of age, female wild type (*Gramd1c* ^+/+^) or *Gramd1c* knockout mice (*Gramd1c* ^−/−^) were switched from standard rodent chow to one of two experimental synthetic diets containing low (0.02%, wt/wt) or high (0.2%, wt/wt) levels of dietary cholesterol. Mice were maintained on these diets over a 4-week period of study, and thereafter bulk RNA sequencing was performed to identify differentially-expressed genes. Data are presented as mean ± SEM from n = four to five mice per group. * = significantly different (*p* ≤ 0.05) when comparing *Gramd1c* ^+/+^ and *Gramd1c* ^−/−^ mice within each diet group.

We next performed bulk RNA sequencing to understand the effects of *Gramd1c* deletion on global gene expression in the liver (Figure 9). The most differentially expressed gene was *Gramd1c*, which was markedly reduced in *Gramd1c* ^−/−^ mice compared to wild type *Gramd1c* ^+/+^ controls in both low and high cholesterol diet conditions (Figure 9). It is important to note that several sterol-responsive genes including proprotein convertase subtilisin/kexin type 9 (*Pcsk9*), squalene epoxidase (*Sqle*), low density lipoprotein receptor (*Ldlr*), 3-hydroxy-3-methylglutaryl-CoA synthase 1 (*Hmgcs1*), and 7-dehydrocholesterol reductase (*Dhcr7*) were significantly repressed under high dietary cholesterol conditions, but the dietary cholesterol-driven suppression of these genes was similar in both *Gramd1c* ^+/+^ and *Gramd1c* ^−/−^ mice (Figure 9). Unexpectedly, the expression of two mitochondrially-encoded genes, mitochondria encoded tRNA-Ile (AUU/C) (*Mt-Ti*) and mitochondrially-encoded tRNA-Gln (CAA/G) (*Mt-Tq*), were elevated in *Gramd1c* ^−/−^ mice only under low dietary cholesterol conditions (Figure 9). In parallel, only under low dietary cholesterol conditions, the expression of several members of the cytochrome P450 family 3 subfamily A genes (*Cyp3a41a*, *Cyp3a41b*, *Cyp3a44*, and *Cyp3a16*) were significantly lower in *Gramd1c* ^−/−^ mice when compared to *Gramd1c* ^+/+^mice (Figure 9). Also, the hepatic expression of several genes encoding major urinary proteins (*Mup7*, *Mup11*, *Mup12*, and *Mup20*) were elevated in *Gramd1c* ^−/−^ mice, only under low dietary cholesterol conditions (Figure 9). Other genes altered in low cholesterol-fed *Gramd1c* ^−/−^ mice includes solute transport genes (*Slc22a28* and *Slc41a2*), sulfotransferase family 3A, member 2 (*Sult3a2*), and sushi domain-containing 4 (*Susd4*). These data show that, particularly under low dietary cholesterol conditions, *Gramd1c* deficiency is associated with reduced expression of several genes involved in xenobiotic metabolism (*Cyp3a41a*, *Cyp3a41b*, *Cyp3a44*, *Cyp3a16, and Sult3a2*) as well as increased expression of major urinary protein genes (*Mup7*, *Mup11*, *Mup12*, and *Mup20*) that encode lipid-binding proteins within the lipocalin family.

### *Gramd1c*-null male mice also have modest alterations in whole body sterol balance

**FIGURE 10:**
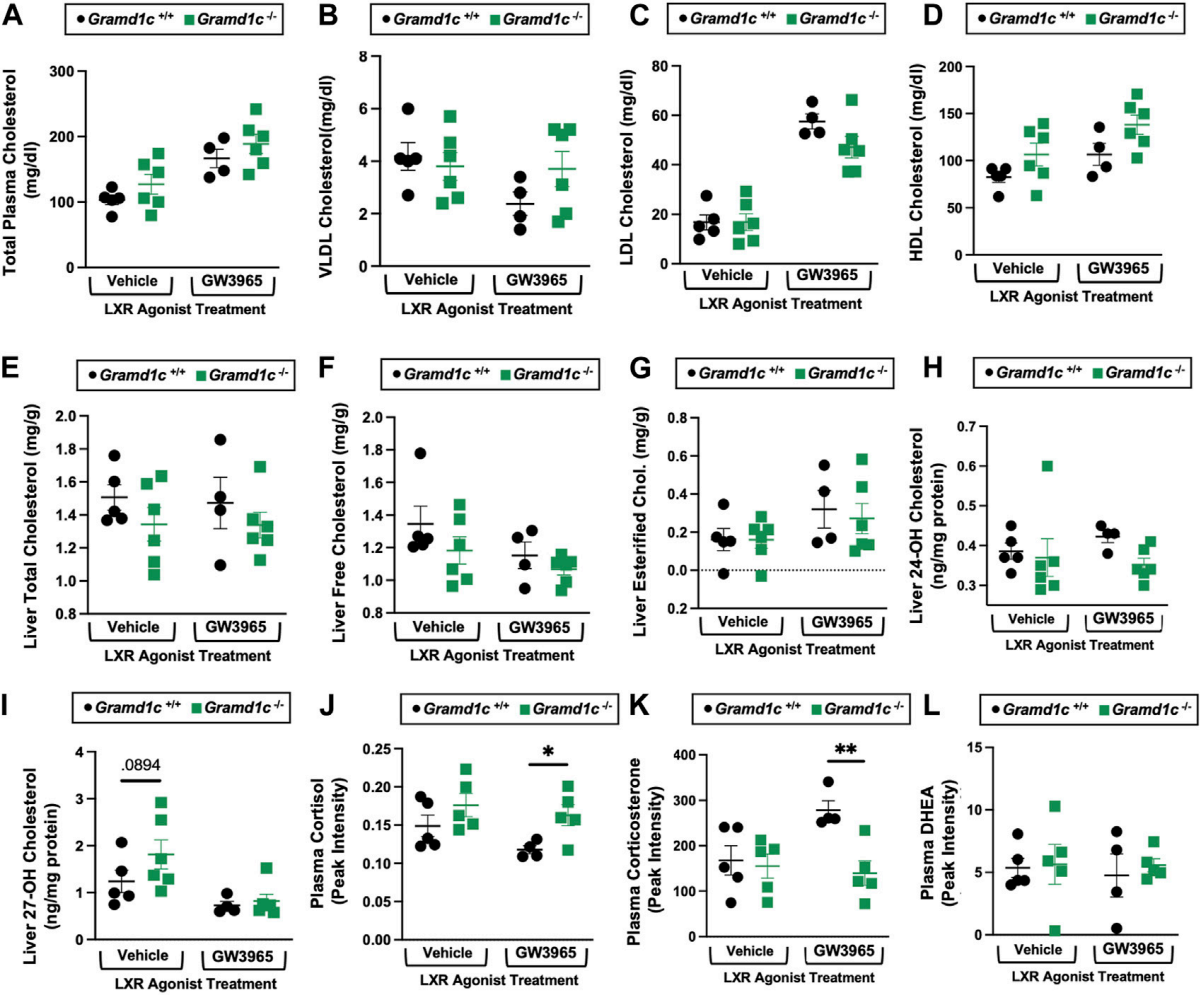
Global deletion of *Gramd1c* does not significantly alter circulating or hepatic cholesterol levels in male mice. At 8 weeks of age, male wild type (*Gramd1c* ^+/+^) or *Gramd1c* knockout mice (*Gramd1c* ^−/−^) were maintained on a standard chow diet in the absence (vehicle) or presence of the LXR agonist GW3965 (40 mg/kg per day) for 7 consecutive days. **(A)** Total plasma cholesterol levels were measure by gas chromatography-tandem mass spectrometry (GC-MS/MS). **(B–D)** Plasma was subjected to size exclusion chromatography to examine total cholesterol distribution across very low density (VLDL), low density (LDL), or high density (HDL) lipoprotein fractions. In addition, a variety of hepatic lipids were quantified by targeted mass spectrometry assays including **(E)** liver total cholesterol, **(F)** liver free cholesterol, **(G)** liver esterified cholesterol, **(H)** 24-hydoxycholesterol (24-OH Chol.), and **(I)** 27-hydroxycholesterol (27-OH Chol.) The plasma levels of various steroid hormones including **(J)** cortisol, **(L)** corticosterone, and **(L)** dehydroepiandrosterone (DHEA) were measured semi-quantitatively by liquid chromatography-tandem mass spectrometry (LC-MS/MS). Data are presented as mean ± SEM from n = four to six mice per group. *(*p* ≤ 0.05), **(*p* ≤ 0.01) = significantly different when comparing *Gramd1c* ^+/+^ and *Gramd1c* ^−/−^ mice within each drug treatment group.

Given previous reports (Sandhu et al., 2018) have shown that *Gramd1b* can be a direct transcriptional target of the nuclear hormone receptor liver X receptor (LXR), we next treated male *Gramd1c* ^+/+^ and *Gramd1c* ^−/−^ mice with a pharmacologic LXR agonist (Figure 10). Much like in female mice (Figures 1–9), males lacking *Gramd1c* ^−/−^ had normal plasma and liver cholesterol levels both under vehicle and GW3965-treated conditions (Figures 10A–G). Although there were some modest trends, *Gramd1c* ^−/−^ mice also did not have statistically significant differences in hepatic oxysterol levels (Figures 10H,I). Although *Gramd1c* ^−/−^ mice did not have altered steroid hormone levels under vehicle-treated conditions (Figures 10J–L), upon LXR agonist treatment *Gramd1c* ^−/−^ mice had slightly elevated plasma cortisol levels and reduced levels of corticosterone (Figure 10K) compared to *Gramd1c* ^+/+^ mice. All other steroid hormones measured were not altered in *Gramd1c* ^−/−^ mice (Figure 10L).

## Discussion

It has been nearly 5 years since the original discovery of the Aster protein family as novel regulators of non-vesicular transport of PM cholesterol to the ER (Sandhu et al., 2018). This seminal report unequivocally established that Aster-B plays an essential roles in cholesterol balance and steroid hormone production, given that *Gramd1b* ^−/−^ mice have very low levels of cholesteryl ester storage in the adrenal gland (Sandhu et al., 2018). However, the cell autonomous and systemic roles of Aster-A and Aster-C in cholesterol uptake and metabolism have been more elusive. Given the tissue-specific expression patterns of Aster proteins, and unique nuclear hormone receptor-driven transcriptional control (i.e., LXR-driven transcription of *Gramd1b* and FXR-driven transcription of *Gramd1c*) (Sandhu et al., 2018; Xiao et al., 2023; Ferrari et al., 2023), it is logical to assume that each Aster protein may play unique roles in cholesterol homeostasis. Here, we have specifically addressed the role of Aster-C in tissue and systemic balance. The main findings of this work are: 1) Aster-C does not play a quantitatively important role in determining plasma, liver, or fecal levels of cholesterol itself, cholesterol synthetic intermediates, oxysterols, or phytosterols, 2) Global deletion of *Gramd1c* is associated with reduced deoxycholic acid and increased plasma cortisol, but only in low cholesterol diet-fed settings, 3) *Gramd1c* ^−/−^ mice did not show alterations in sterol-sensitive genes in the liver, but instead showed altered expression of genes in major urinary protein (MUP) and cytochrome P450 (CYP) families only under low dietary cholesterol conditions. Collectively, our results show that Aster-C plays a relatively minor role in whole body sterol balance under conditions where dietary cholesterol is limited or in excess.

Although global *Gramd1c* ^−/−^ mice show a relatively minor phenotype, when compared to the striking adrenal phenotype in *Gramd1b* ^−/−^ mice (Sandhu et al., 2018), a plausible explanation is the potential for functional redundancy of Aster proteins in tissues where several are co-expressed together. For instance, the adrenal gland has relatively high expression of Aster-B compared to Aster-A and Aster-C, and therefore deletion of *Gramd1b* has a profound impact on adrenal cholesterol ester storage (Sandhu et al., 2018). In contrast, the liver expresses all three Gramd1 genes, and there is emerging evidence of functional redundancy. In support of this, findings here show that global deletion of Gramd1c does not alter hepatic sterol homeostasis. However, a recent study by the Tontonoz group (Xiao et al., 2023) demonstrated that hepatocyte-specific deletion of both *Gramd1a* and *Gramd1c* (i.e., hepatocyte-specific *Gramd1a* and *Gramd1c* double knockout) have impaired movement of lipoprotein-derived free cholesterol out of the body via RCT. In further support of functional redundancy, only double knockout of both *Gramd1b* and *Gramd1c* in intestinal enterocytes, but not single deficiency, can reduce intestinal cholesterol absorption (Ferrari et al., 2023). Also, the ability of Chinese hamster ovary (CHO-K1) cells to esterify LDL-derived cholesterol depends on the deletion of all three Aster proteins (A, B, and C), whereas single deletion had modest to no effect (Trinh et al., 2021). Given there is some interest in potentially drugging the Aster proteins for disease of cholesterol imbalance like atherosclerosis and some cancers (Xiao et al., 2021), it will be imperative to understand where functional redundancy exists and those situations where one family member can be selectively targeted for therapeutic benefit.

Although there is mounting evidence of functional redundancy for Asters, further studies are needed to understand where certain family members predominate. For example, it is clear that LXR-driven expression of Aster-B is essential for the esterification and storage of cholesterol esters in the adrenal gland, and that Aster-A and -C cannot combine to overcome the loss of Aster-B (Sandhu et al., 2018). Here we show that under conditions of global Aster-C deficiency, there is clearly significant reduction in the major bile acid species deoxycholic acid (Figure 7A). Given recent reports showing that *Gramd1c* is a direct transcriptional target of the bile acid sensing nuclear receptor FXR (Xiao et al., 2023), additional studies are needed to further understand whether Aster-C-driven modulation of bile acid homeostasis may hold therapeutic potential in liver disease (Wang et al., 2020). In addition to cholesterol transport, Aster proteins have also been shown to transport carotenoids in the retina (Bandara et al., 2022). It is interesting to speculate whether diverse Aster proteins including Aster-C may play a broader role in lipid transport and metabolism beyond sterols and carotenoids. Of potential interest in this study, the clear upregulation of the hepatic major urinary protein (MUP) genes (*Mup7*, *Mup11*, *Mup12*, and *Mup20*) may be worth further exploration. MUP proteins are lipid-binding proteins within the lipocalin family, and are known to carry diverse lipid cargos in the bloodstream and urine for the purpose of chemical signaling (Zhou and Rui, 2010). Additional work is clearly needed to fully understand the specific role of Aster-C in lipid transport and metabolism. In conclusion, this work shows that Aster-C plays a minor role in whole body sterol homeostasis. However, additional work is needed to determine whether this is simply due to functional redundancy of other Aster proteins or indicative of other elusive functions of Aster-C in lipid trafficking. Naito et al., 2023.

## Data Availability

The datasets presented in this study can be found in online repositories. The names of the repository/repositories and accession number(s) can be found below: https://www.ncbi.nlm.nih.gov/genbank/, GSE250230.
